# THE HEALTHIER BLACK ELDERS CENTER COMMUNITY-ENGAGED PROGRAM

**DOI:** 10.1093/geroni/igae098.0728

**Published:** 2024-12-31

**Authors:** Peter Lichtenberg, Tam Perry, Vanessa Rorai, LaToya Hall

**Affiliations:** Wayne State University, Detroit, Michigan, United States; Wayne State University, Detroit, Michigan, United States; Healthier Black Elders Center, Detroit, Michigan, United States; Wayne State University, Detroit, Michigan, United States

## Abstract

For the past 25-years the Healthier Black Elders Center has been the community-engaged program run by the Wayne State University Institute of Gerontology as part of the Michigan RCMAR program. The HBEC has an active Community Advisory Board and engages in year round community education and health programs, provides a lay person newsletter and has a Participant Registry. During the past five years the HBEC began a community-engaged consultancy program in order to assist others across the country in engaging with older black adults for research participation. The HBEC is also a science generating program, with research publications across the past 20 years on recruitment and retention of older black adults into research. Beginning in 2012 the HBEC also expanded to include the Successful Aging through Financial Empowerment program. The SAFE program has been the nexus for financial-decision making, financial exploitation and day to day financial management in older black adults. The SAFE program is not only a research program but also a service program. The SAFE program provides 1:1 assistance for older adults who were victims of scams or identity theft and are having difficulty straightening out their finances. In order to reach older black adults and the professionals who work with them the SAFE program has its own year round community engaged efforts including the program “Financial First Fridays” and a regular series of community-based talks. SAFE has assisted over 350 older adults and caregivers and provided 1:1 services for 200 clients during the past five years.

